# Ointment of *Ximenes americana* promotes acceleration of wound healing in rats^1^

**DOI:** 10.1590/s0102-865020190030000007

**Published:** 2019-03-18

**Authors:** José de Castro Souza, Lígia Reis de Moura Estevão, Adriana Aparecida Ferraz, Ricardo Santos Simões, Marcela Gabriela Feitosa Vieira, Joaquim Evêncio-Neto

**Affiliations:** I Fellow PhD degree, Postgraduate Program in Animal Bioscience, Department of Morphology and Animal Physiology, Universidade Federal Rural de Pernambuco (UFRPE), Recife-PE, Brazil. Acquisition and interpretation of data, technical procedures, histopathological examinations, statistics analysis, manuscript preparation.; II PhD, Department of Morphology and Animal Physiology, UFRPE, Recife-PE, Brazil. Acquisition, analysis and interpretation of data; manuscript writing.; III PhD, Department of Morphology and Genetics, Universidade Federal de São Paulo (UNIFESP), Brazil. Histopathological examinations, manuscript preparation, critical revision.; IV PhD, Department of Morphology and Genetic, UNIFESP, Sao Paulo-SP, Brazil. Analysis of data, manuscript writing, final approval.; V Fellow Master degree, Department of Morphology and Animal Physiology, UFRPE, Recife-PE, Brazil. Analysis and interpretation of data, manuscript writing.; VI Full Professor, Department of Morphology and Animal Physiology, UFRPE, Recife-PE, Brazil. Conception and design of the study, manuscript preparation, critical revision.

**Keywords:** Wound Healing, Phytotherapy, Fibroblast, Collagen, Skin, Rats

## Abstract

**Purpose:**

To evaluate the healing potential of the *Ximenia americana* hydroalcoholic extract in 10% cream in excisional wound models in rats.

**Methods:**

Sixty male adults Wistar rats were submitted to skin and subcutaneous tissue surgery in the right and left thoracic regions, divided into three experimental groups: Standard submitted to treatment with only the base vehicle, Treated wounds treated with hydroalcoholic extract of X. americana applied on 10%, Lanette base and Control, untreated wounds. The treatment was performed daily and the wounds evaluated microscopically by the quantification of fibroblasts, collagen fibers and blood vessels.

**Results:**

The histomorphometric analysis showed a significant increase in the number of fibroblasts, collagen fibers and blood vessels in the treated group.

**Conclusion:**

The topical action of the cream based on *Ximenia americana* shows angiogenic effects and improves the replacement of collagen, suggesting its use for the development of herbal remedy in the treatment of cutaneous wound healing.

## Introduction

 Since ancient times man uses products derived from plants with curative power, and nowadays most of the medicines used had the same origin and principle ^1^. Thus, due to the enormous biodiversity of native species present in northeastern Brazil there is one that we tried to evaluate because it is normally used by the population^1^ ^,^ ^2^. Besides this it could also be used as a potential source for the development of new natural products, among which the *Ximenia americana* (X. americana), popularly known as yellow plum, wild plum or sea lime^2^. The use of this species has demonstrated strong curative power due to the presence of considerable bioactive compounds related to antioxidant activity, tannins and flavonoids^1^ ^,^ ^3^. According to Souza *et al.* ^1^ and Almeida *et al.* ^3^, spraying produced of the plant bark is used in folk medicine in the healing of ulcers, as an anti-inflammatory activity. It can still be used as a depurative, menstrual regulator and in gastric disorders. The infusion of its flowers is also used against diarrhea. It is also commonly used in insect bites and has antipyretic activity^4^ ^-^ ^7^.

 The chemical composition of its secondary metabolites is broad. The stem and roots of this plant contain saponins, glycosides, flavonoids, tannins, phenolics, alkaloids, types of quinones and terpenoids. In addition, the plant is rich in fatty acids and glycerides and the seeds contain cyanide derivatives^1^ ^,^ ^8^. The microbial activity of X. americana is attributed to tannins and terpenoids^4^. Sobeh *et al.* ^9^ suggest that the tannin-rich extract of X americana works in the treatment of various health disorders associated with oxidative stress, such as hepatocellular injury and diabetes. Polyphenols have antiallergic, antibacterial, antifungal, anti-inflammatory and vasopressor effects^10^ ^-^ ^12^. Nevertheless, it was not found studies describing fibroblasts and cicatrization associate to X. americana extract.

 The repair of cutaneous wounds is essential to life and the faster the resolution occurs, the shorter the time exposure of the organism to a series of pathogens^13^ ^,^ ^14^. Considered a dynamic process of biochemical and physiological phenomena, cicatrization develops through cellular and molecular events, which interact in a coordinated way to ensure cell restoration and constitution^15^. In general, the healing process follows a pattern and can be divided into three phases: inflammatory, fibroblastic and remodeling^16^. The inflammatory phase begins immediately after the injury and consists of a vascular response (hemostasis) and a cellular response, with a differentiation, proliferation and migration of cells for tissue recovery^14^ ^,^ ^17^.

 The fibroblast phase begins around the fourth day after the injury and goes through the end of the second week. It can be divided into four fundamental steps: epithelization, angiogenesis, formation of granulation tissue and collagen deposition. During this phase, the migration and proliferation of fibroblasts occurs, at the same time, as the synthesis of a new extracellular matrix components is performed^18^ ^,^ ^19^. Neovascularization occurs in parallel with the fibroplasia process and is essential at this stage because it allows the exchange of gas and nutrition from metabolically active cells. In addition to the direct action of growth factors, especially VEGF (vascular endothelial growth factor) on vessel endothelial cells, angiogenesis induction is also influenced by the low oxygen tension that occurs at the center of a wound^20^.

 The last stage of the healing process is the remodeling phase, which can occur for months or even years. At this stage there is gradual reduction of inflammatory cells and ceases angiogenesis and fibroplasia. It is also during this period that the balance between synthesis and degradation of collagen is observed, and this remodeling is responsible for the increase in the tensile strength of scar tissue^18^.

 This work had the objective of evaluating the potential healing of X. american extract cream on cutaneous wounds. Based on positive results with X. americana in popular medicine, as its chemical constituents and antimicrobial action were reported, and this study also aimed to make a cream manipulation in base and sufficient concentration to favour the process of tissue repair.

## Methods

 This study was approved by the Research Ethics of Animal of Universidade Federal Rural de Pernambuco - UFRPE (process nº 088/2015).

 Sixty adult male rats (*Rattus* *norvegicus* *albinus*), ±300g, from the Department of Morphology and Physiology of the UFPE were used in this experiment and the care and procedures used such as: anesthesia,trichotomy,preparation of the *Ximenia americana* branch extract,preparation of cutaneous lesions, as well as treatment of groups such as ointment application were already described in Castro Souza *et al.* ^21^

The experimental design was completely randomized and equally divided into three groups (20 animals each one), as follow: GX - wounds treated with 10% branch extract of X. americana; GP - wounds treated with vehicle; GC - animals with untreated wounds. Each wound was treated immediately after surgery, and daily as described by the following methodology: the treated group, daily topical application of X. americana branch extract, in sufficient quantity to cover the wound; default group, daily topical application of Lanette base cream and control group, received only management similar to the other groups, but the wounds were not treated. Five animals from each group were evaluated on 4, 7, 14 and 21 days after surgery. Euthanasia was performed by deepening of anesthesia (xylazine - 20 mg/Kg and ketamine - 100 mg/Kg, intramuscularly).

### Morphology and morphometry

 On 4th, 7th, 14th and 21st days after surgery, the animals anesthetized as previously described, each surgical wound were dissected with 0.5 cm margin of integral whole skin on the perimeter of the lesion and depth to the dorsal muscular fascia. Then, the collected wound specimens were processed for histological and morphometrical analysis.

### Histological and morphometric analysis

 After this, the fragments were fixed in 10% formaldehyde, tissue samples were then processed for paraffin inclusion. For each animal cuts were made in the middle region of the flap sections longitudinal samples (5µm) were obtained parallel to the greater axis of fragments and stained with Hematoxylin and Eosin (H.E) and Masson’s Trichrome for analysis of the density of collagen, number of blood vessels and fibroblasts.

 To determine the density of fibroblasts, blood vessels and collagen fibers it was performed in the center of the lesion in an area of 0.66 mm2 (imaging), according to the method standardized by Kamp *et al.* ^22^ with the aid of an image analyzer (by Image Pro-plus^®^ software) in a Windows operational system. Six images of each slide stained with Masson’s Trichrome was obtained, with the aid of a trinocular biological microscope (NIKON 50i) under x400 magnification and adjusted to a capturing image system.

### Statistical analysis

 The data was evaluated by non-parametric Kruskal-Wallis test, between each variable and among the 4, 7, 14 days for fibroblasts, collagen fibers and blood vessels quantification. In the cases that significant differences occurred among the groups or among the days, the Dunn test was applied (p<0.05). Statistical analysis was performed using the Statistical Package for the Social Sciences - SPSS 20^®^ software.

## Results 

 The phytochemical data of the hydroalcoholic extract of *Ximenia americana* in Thin Layer Chromatography (CCD) are shown in Table 1. The phytochemical study of the hydroalcoholic extract of *Ximenia americana* in Thin Layer Chromatography (CCD) revealed a chemical classification with the presence of tannins, flavonoids and terpenoids. Reactions for the characterization of alkaloids, steroids, anthraquinone and coumarin were negative for these constituents.

**Table 1:** Phytochemical prospecting of the hydroalcoholic extract of Ximenia Americana.

**Chemical class**	**Revelation technique**	**Hydroalcoholic extract**
Tannins	Ferric chloride	**+**
Alkaloids	Dragendorff	**-**
Flavonoids	NP-PEG / Aluminum chloride / serum sulfate	**+**
Terpenoids	Liberman anisaldehyde	**+**
Steroids	Liberman anisaldehyde	**-**
Anthraquinones	Potassium hydroxide	**-**
Coumarin	Potassium hydroxide	**-**

**Figure 1:**
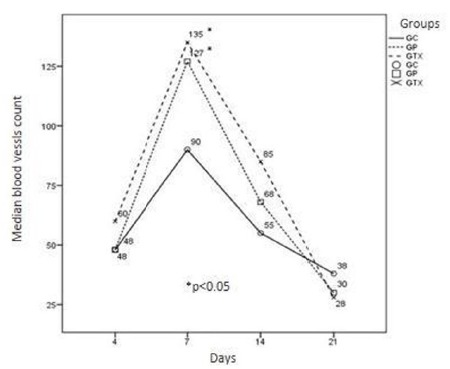
Median of blood vessels in the control (GC), standard (GP) and X. americana (GTX) groups, on postoperative days 4, 7, 14 and 21. A significant difference was observed in the number of vessels in the X. americana (GTX) group (p <0.05), when compared to the groups (GP and CG), during the same period, on day 7 when compared to each other days (p <0.05).

**Figure 2:**
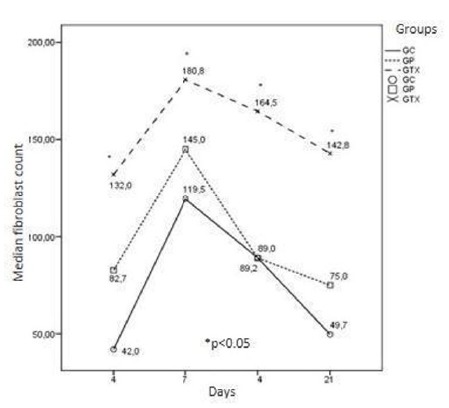
Median of fibroblasts count in the groups treated with *Ximenia Americana* (GTX), standard group (GP) and control group (CG), on postoperative days 4, 7, 14 and 21. A significant difference in the amount of fibroblasts from the X. americana (GTX) - treated group was observed when compared with the standard (GP) and control (CG) groups (p <0.05).

**Figure 3:**
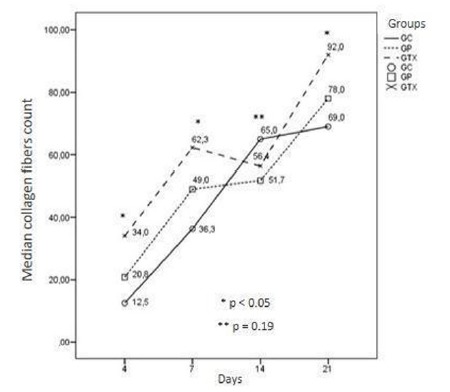
Median of collagen fibers in the groups treated with X.americana (GTX), standard group (GP) and control group (CG), on postoperative days 4, 7, 14 and 21. A significant difference in the amount of collagen fibers from the X. americna treated group (GTX) (p <0.05) was observed when compared to the standard (GP) and control (CG) groups, on postoperative days 4, 7 and 21 , on day 14 the difference is not statistically significant (p = 0.19).

 Our morphological data showed higher concentration of blood vessels, fibroblasts and organization of collagen fibers in the groups treated with *Ximenia americana* at all study ages (Figs. 1 to 3). 

## Discussion

 Our results in thin layer chromatography (CCD) showed that the hydroalcoholic extract of X. americana is rich in tannins, flavonoids and terpenoids, and the reactions for the characterization of alkaloids, steroids, anthraquinone and coumarin were negative. The quantification of tannins by the Folin-Ciocalteu method showed the presence of high total phenol content by the spectrophotometric assay, in agreement with the findings of Brasileiro *et al.* ^23^. Constituent of the secondary metabolism of several plants the phenolic compounds tannins and flavonoids stand out for having antioxidant, anti-inflammatory and healing action. It is known that tannin protects the wound by increasing the thickness of the crust, besides favoring hemostasis by precipitating proteins and possessing antimicrobial action, whereas flavonoids act as antioxidants and present antiinflammatory, regenerative, antimicrobial and modulatory action of the immune system^24^. The determination of the amount of total phenols of the X. americana extract is of fundamental importance since these phenolic compounds, mainly tannins and flavonoids, are attributed to antioxidant effects^25^ ^,^ ^26^.

 The bases of creams are commonly indicated for the incorporation of extracts in the most varied applicability, among them the Lanette anionic base, which is one of the oldest and most used, made from Lanette cream (cetostearyl alcohol and cetyl stearyl sulfate). The Lanette base is preferred for imparting significant stability to the product^21^. Considering the medical/cosmetic interest the cream can not be irritating, it should not degrade easily and must be compatible with the active principles and with the special additives^27^. The choice of base to be used in the formulation depends on the influence of the drug on the consistency or other properties of the base, as well as on the stability of the drug in the base^28^.

 Based on Brasileiro *et al.* ^23^ findings, when studying new semi-solid dosage forms for *Ximenia americana*, the hydrocoholic extract does not stabilize on an ointment basis, despite maintaining the odor and dark brown color characteristic of the extract. The Lanette cream preparations, used in this experiment, presented homogeneous appearance throughout the stability study, maintaining the same characteristic odor and color^21^.

 During the repair phase granulation tissue formation occurs, with proliferation of blood vessels (endothelial cells) and fibroblasts. Neovascularization, is important in the proliferation process, begins with capillary formation resulting from the release of angiogenic factors secreted by macrophages that stimulate the proliferation of endothelial cells. This neovascularization of the region occurs in parallel with the process of fibroplasia that is essential at this stage because it allows gas exchange and nutrition of the metabolically active cells^19^ ^,^ ^20^.

 Our results showed a significant increase in fibroblasts and blood vessels in the treated group (p <0.05) when compared to GP and GC groups at all times studied. In addition, a significant difference was observed in the density and arrangement of the collagen fibers of the *Ximenia americana* treated group in relation to the standard and control groups.

 This significant increase in the number of collagen fibers corroborates the results found by Carvalho *et al.* ^29^ when using 20% aqueous X. americana extract in mouse wounds. Similar findings were reported by Brasileiro *et al.* ^23^ when they affirm that there is more extracellular deposition of collagen in the cutaneous wound of rats treated with X. americana cream, accelerating the cicatricial process. Leal *et al.* ^27^ demonstrated high efficiency of Ximenian ethanolic extract gel associated with phonophoresis in the proliferation of fibroblasts and biomechanical properties of the tendon in rats. According to Moura *et al.* ^30^, collagenation of a wound represents one of the most significant factors for the dermal recovery after the aggression. The amount of collagen in the wound increases as the days go by. About two weeks the fibers predominate in the extracellular environment.

## Conclusion

 The topical action of the cream based on *Ximenia americana* shows angiogenic effects and improves the replacement of collagen, suggesting its use for the development of herbal remedy in the treatment of cutaneous wound healing.
